# Comparison of Postoperative Renal Function between Non-Steroidal Anti-Inflammatory Drug and Opioids for Patient-Controlled Analgesia after Laparoscopic Nephrectomy: A Retrospective Cohort Study

**DOI:** 10.3390/jcm9092959

**Published:** 2020-09-13

**Authors:** Jiwon Han, Young-Tae Jeon, Ah-Young Oh, Chang-Hoon Koo, Yu Kyung Bae, Jung-Hee Ryu

**Affiliations:** 1 Department of Anesthesiology and Pain Medicine, Seoul National University Bundang Hospital, 82, Gumi-ro, Bundang-gu, Seongnam-si, Gyeonggi-do 13620, Korea; hanjiwon@snubh.org (J.H.); ytjeon@snubh.org (Y.-T.J.); ohahyoung@hanmail.net (A.-Y.O.); vollock9@gmail.com (C.-H.K.); vansuri@naver.com (Y.K.B.); 2 Department of Anesthesiology and Pain Medicine, Seoul National University College of Medicine, 103, Daehak-ro, Jongno-gu, Seoul 03080, Korea

**Keywords:** GFR, nephrectomy, NSAID, opioid, PCA

## Abstract

Non-steroidal anti-inflammatory drugs (NSAIDs) can be used as opioid alternatives for patient-controlled analgesia (PCA). However, their use after nephrectomy has raised concerns regarding possible nephrotoxicity. This study compared postoperative renal function and postoperative outcomes between patients using NSAID and patients using opioids for PCA in nephrectomy. In this retrospective observational study, records were reviewed for 913 patients who underwent laparoscopic or robot-assisted laparoscopic nephrectomy from 2015 to 2017. After propensity score matching, 247 patients per group were analyzed. Glomerular filtration rate (GFR) percentages (postoperative value divided by preoperative value), blood urea nitrogen (BUN)/creatinine ratios, and serum creatinine percentages were compared at 2 weeks, 6 months, and 1 year after surgery between users of NSAID and users of opioids for PCA. Additionally, postoperative complication rates, postoperative acute kidney injury (AKI) incidences, postoperative pain scores, and lengths of hospital stay were compared between groups. Postoperative GFR percentages, BUN/creatinine ratios, and serum creatinine percentages were similar between the two groups. There were no significant differences in the rates of postoperative complications, incidences of AKI, and pain scores at 30 min, 6 h, 48 h, or 7 days postoperatively. The length of hospital stay was significantly shorter in the NSAID group than in the opioid group. This study showed no association between the use of NSAID for PCA after laparoscopic nephrectomy and the incidence of postoperative renal dysfunction.

## 1. Introduction

Postoperative pain control with patient-controlled analgesia (PCA) enhances the quality of recovery of patients. However, there are risks associated with opioid analgesic over- or under-doses depending on the individual patient’s needs or the type of surgery and drug-related side effects [1,2]. The selection of PCA drugs and doses with sufficient effects, while minimizing the side effects, is an important responsibility for anesthesiologists. Thus far, PCA regimens have primarily used opioids or opioids plus adjuvant drugs [3]. Opioid-based PCA has adverse effects, including respiratory depression, nausea, vomiting, sedation, pruritus, and urinary retention. Therefore, the development of an opioid alternative as PCA is important [4].

Non-steroidal anti-inflammatory drugs (NSAIDs) are widely used for postoperative pain control and can be considered opioid alternatives for PCA. NSAID PCA is tolerable for postoperative pain control and facilitates better bowel function recovery [5,6]. NSAIDs also have adverse effects such as gastrointestinal ulceration, increased risk of perioperative bleeding, thromboembolic events, and nephrotoxicity [7,8,9,10,11]. The nephrotoxicity mechanism involves the cyclooxygenase blockade of NSAIDs, which subsequently inhibits the synthesis of prostaglandin, a renal vasodilator. This NSAIDs effect reduces renal blood flow and the glomerular filtration rate (GFR) [8]. A previous study showed that long-term use of ketorolac, an intravenous NSAID, is associated with an increased incidence of acute renal failure [12]. Therefore, clinicians have expressed concerns regarding the use of NSAIDs in patients undergoing nephrectomy, and there remains controversy about safety. A few previous investigations have shown no association between the use of ketorolac and renal dysfunction [13,14]. However, a prior study showed that the use of ketorolac was a potential risk factor for long-term renal dysfunction in kidney donors [15]. 

In our institution, we have used both NSAID and opioids PCA for many years in patients undergoing laparoscopic nephrectomy, in accordance with the preferences of anesthesiologists and surgeons. In this retrospective analysis, we compared the effects of NSAID PCA on renal function with those of opioid PCA. The primary outcome of our study was the GFR after laparoscopic nephrectomy; secondary outcomes included renal function test results, rates of postoperative complication, incidences of acute kidney injury (AKI), pain scores, and length of hospital stay.

## 2. Materials and Methods

### 2.1. Study Design and Patients

This retrospective observational study was approved by the Institutional Review Board of Seoul National University Bundang Hospital (approval number: B-1909/565-103). The requirement of informed consent was waived. The data were based on the electronic medical records of patients who underwent laparoscopic or robot-assisted laparoscopic nephrectomy between January 2015 and December 2017. Demographic data, preoperative co-morbidity, anesthetic and operative data, renal function test including GFR, blood urea nitrogen (BUN), and serum creatinine before and after surgery for up to one year postoperatively were collected. Additionally, rates of postoperative complication, pain scores, and length of hospital stays were also collected.

### 2.2. Anesthesia, Surgical Procedure, and PCA Regimen

All patients underwent balanced general anesthesia. The main anesthetic agents were desflurane, sevoflurane, or propofol continuous infusion. The adjuvant analgesic agent was remifentanil using target-controlled infusion, and the neuromuscular blocking agent was rocuronium. Pulse oximetry, non-invasive blood pressure, invasive arterial blood pressure, electrocardiography, capnography, and esophageal temperature were monitored. Patients were placed in the lateral decubitus position. Pneumoperitoneum was made with a Veress needle technique, then the 12 mm camera port was placed, and the other ports were placed under the vision of a laparoscope. All of the surgical procedures were performed with a transperitoneal approach. The colon was dissected medially from the Toldt’s line, and the Gerota’s fascia was opened. After identification of the ureter, renal artery, and vein, the kidney was mobilized. The renal artery and vein were clamped using laparoscopic bulldog clamps, and the tumor or kidney was resected. The specimen was inserted into an endocath bag and retrieved through the camera port. A Jackson–Pratt drain was inserted. After closing the wound, aseptic dressing or surgical skin glue was applied. At the end of the surgery, intravenous PCA was initiated. NSAID PCA was made of ketorolac, with 3.6 mg of bolus, 3.6 mg of continuous basal infusion per hour, a lock out time of 15 min, and a total volume of 50 mL. Opioid PCA was made of fentanyl or oxycodone, and the doses varied with age and weight: bolus of fentanyl was 5 µg to 21 µg, with 5 µg to 21 µg of continuous basal infusion per hour; bolus of oxycodone was 0.4 mg to 1.2 mg, with 0.4 mg to 1.2 mg of continuous basal infusion per hour, and the lock out time was 15 min with a total volume of 100 mL. Immediately before using opioid PCA, 0.3 mg of ramosetron or 0.075 mg of palonosetron was administered as a prophylactic antiemetic agent. If a PCA refill was required, the NSAID group was refilled with fentanyl in consideration of age and weight, and the opioid group was refilled with the opioid in the same way as the initial dose.

### 2.3. Outcomes

The primary endpoint was postoperative GFR at 2 weeks, 6 months, and 1 year after laparoscopic nephrectomy. GFR was compared by GFR percentage, defined as postoperative GFR divided by preoperative GFR. GFR was calculated by using the CKD-EPI (Chronic Kidney Disease Epidemiology Collaboration) equation, which had less bias and greater accuracy to the GFR value from serum creatinine [16]. The subgroups were divided into radical and partial nephrectomy to reduce the bias following surgery type, and GFR at 2 weeks, 6 months, and 1 year after surgery were compared between the NSAID and opioid groups. Secondary endpoints were the renal function test results, rates of postoperative complication defined by Clavien–Dindo classification, incidences of postoperative acute kidney injury (AKI), pain scores measured by an 11-point numerical rating scale (NRS) at 30 min, 6 h, 24 h, 48 h, and 7 days after surgery, and length of hospital stays. The renal function test included BUN/creatinine ratio and creatinine percentage, defined as postoperative creatinine divided by preoperative creatinine. The Kidney Disease: Improving Global Outcomes (KDIGO) criteria were used to define and grade the AKI [17]. However, considering Foley catheter use after nephrectomy, only serum creatinine was used for defining and grading AKI. AKI stage 1 was defined as an increase in serum creatinine by 0.3 mg/dL, or 1.5 to 1.9 times that of the preoperative level. AKI stage 2 was defined as an increase in serum creatinine by 2.0 to 2.9 times that of the preoperative level. AKI stage 3 was defined as an increase in serum creatinine by 4.0 mg/dL, or more than three times that of the preoperative level, or initiation of renal replace therapy.

### 2.4. Statistical Methods

A propensity score matching method was performed to balance the covariates between the groups. The following covariates—age, sex, weight, height, and anesthetic time—were matched at a 1:1 ratio with the nearest neighbor methods using MatchIt package on R 3.6.1 (R Project for Statistical Computing, Vienna, Austria) [18]. Patient demographics and primary and secondary outcomes were compared using the χ^2^ test for categorical variables and the Student’s *t*-test for continuous variables. Statistical significance was considered at a *p*-value of less than 0.05. All statistical analyses were performed using SPSS software version 25 (SPSS Inc., Chicago, IL, USA).

## 3. Results

**Figure 1:**
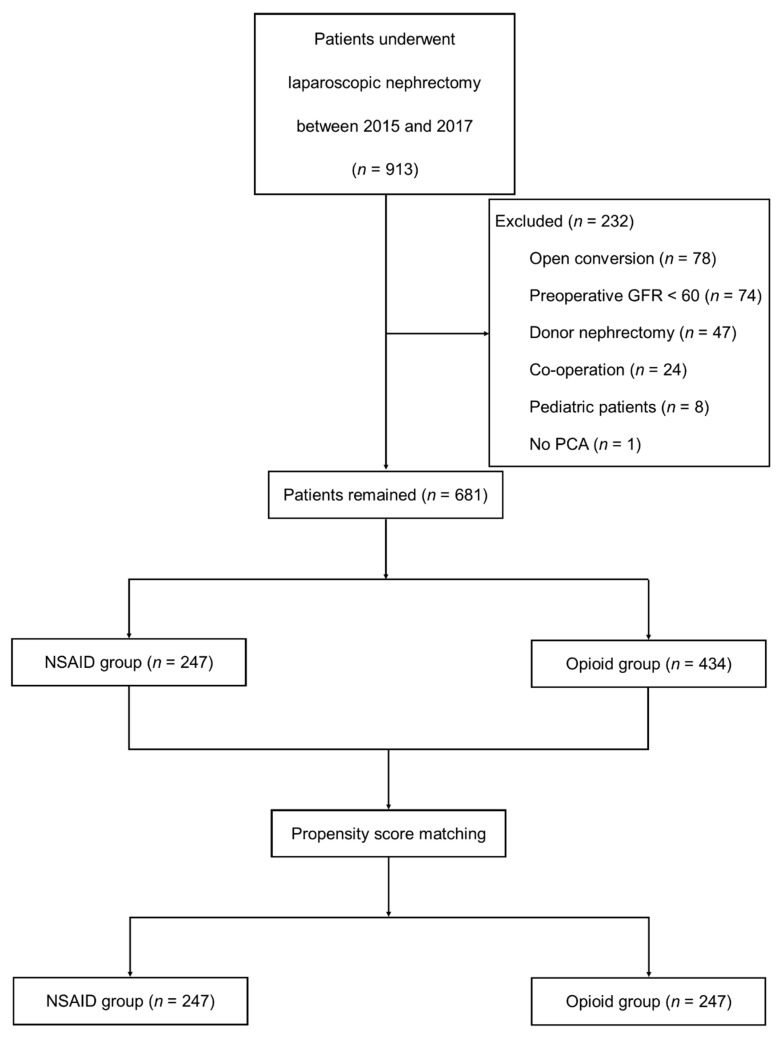
Retrospective study design and study flow diagram. NSAID, non-steroidal anti-inflammatory drug; PCA, patient-controlled analgesia.

A total of 913 patients were identified from the database. Two hundred thirty-two patients were excluded: 78 patients who underwent open nephrectomy, 74 patients with a preoperative GFR of below 60 mL/min/1.73 m^2^, 47 patients who were kidney donors, 24 patients who underwent nephrectomy with other surgeries, 8 patients who were under the age of 20, and 1 patient who did not use PCA. The remaining 681 patients were included for analysis (Figure 1). After propensity score matching, 247 patients remained in each group. Table 1 shows patient demographic data and preoperative renal function test results of the two groups after propensity score matching. Table 2 presents anesthetic and surgical data of the patients. After propensity score matching, this study showed that all covariates were similar between the two groups, except the cancer resection margin. The resection margin was cancer-free in all patients, but the length of the resection margin was greater in patients using opioids than in patients using NSAID. Most patients had renal cell carcinoma. Neoplasms other than renal cell carcinoma included angiomyolipoma, oncocytoma, mixed epithelial tumor, mesenchymal tumor, and complicated renal cyst. Other disorders included ureteropelvic junctional obstruction, gangrenous pyelonephritis, and painful atrophic kidney.

**Figure 2:**
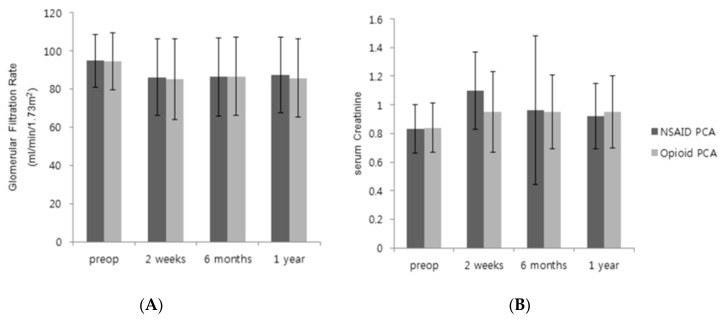
Pre- and postoperative glomerular filtration rate (GFR) and serum creatinine. (**A**) GFR, (**B**) serum creatinine. Comparison of GFR and serum creatinine between NSAID PCA group and opioid PCA group. There were no significant differences. Results are expressed as mean ± SD.

**Figure 3:**
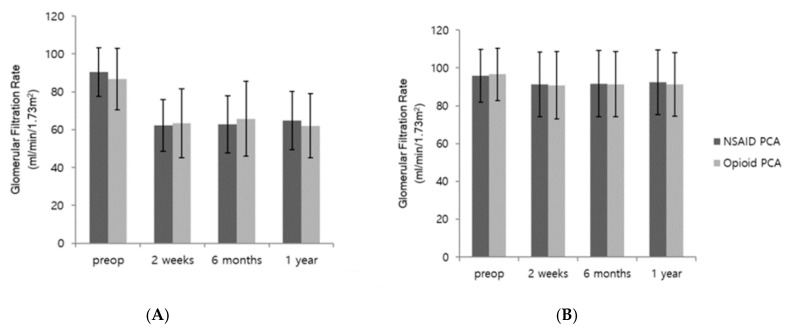
Subgroup analysis of glomerular filtration rate. (**A**)**:** Radical nephrectomy, (**B**)**:** Partial nephrectomy. Comparison of GFR between NSAID PCA group and opioid PCA group. There were no significant differences in both radical and partial nephrectomy. Results are expressed as mean ± SD.

The primary and secondary outcomes are shown in Table 3 and Figure 2. The GFR percentages at 2 weeks, 6 months, and 1 year after laparoscopic nephrectomy were comparable between groups. Figure 2 presents the pre- and postoperative GFRs and creatinine levels, which were similar between the two groups. The BUN/creatinine ratios and creatinine percentages were also similar between the two groups. The stages of Clavien–Dindo complications, incidences of postoperative AKI, and postoperative pain scores at 30 min, 6 h, 48 h, and 7 days after surgery were also similar between the two groups. However, the pain scores at 24 h postoperatively were lower (3.8 vs. 4.0, *p* = 0.01), and the numbers of PCA-use days were smaller in the NSAID group (3.2 vs. 3.9, *p* < 0.001) compared with the opioid group. Moreover, the length of hospital stay was significantly shorter in the NSAID PCA group than in the opioid PCA group (7.5 vs. 8.5 days, *p* < 0.001). Figure 3 showed that our subgroup analyses revealed no differences in GFRs between NSAID and opioid groups in patients who underwent radical (Figure 3A) or partial nephrectomy (Figure 3B).

## 4. Discussion

The present retrospective observational study compared the renal functions and postoperative outcomes, including complication rate, incidence of AKI, pain scores, and length of hospital stay, between NSAID PCA and opioid PCA groups. This analysis showed similar postoperative renal functions between these two groups after laparoscopic nephrectomy. However, the length of hospital stay was shorter in the NSAID group than in the opioid PCA group.

Subgroup analysis showed that the postoperative GFR reduction was more severe in patients who underwent radical nephrectomy than in those who underwent partial nephrectomy, but no difference was observed between the opioid and NSAID groups. The effect of NSAIDs on the postoperative renal function in our study is consistent with the effects observed in previous nephrectomy investigations [13,14,19]. However, a study by Takahashi et al., which examined patients undergoing live-donor nephrectomy, suggested that NSAIDs may be a risk factor for postoperative renal dysfunction [15]. A possible explanation for this discrepancy may lie in the percentage of patients who underwent partial nephrectomy in our study (80%), given that all patients in the study by Takahashi et al. underwent radical resection [15]. However, as stated above, we found similar GFRs between the NSAID and opioid groups in our subgroup analysis of radical nephrectomy. Therefore, a well-designed prospective study is needed concerning the use of NSAIDs in patients undergoing nephrectomy.

In our study, the incidences of AKI were 9.7% and 7.7% in the NSAID and opioid groups, respectively. A previous study noted an association between administrations of ketorolac for more than 5 days and enhancement of AKI rate [12]. However, in our study, ketorolac was administered for a maximum of 50 h, in accordance with our institution’s PCA regimen guidelines, and the incidence of AKI was not significantly higher in the NSAID group than in the opioid group. The number of PCA-use days was smaller in the NSAID group than in the opioid group because the total PCA volumes were 50 mL and 100 mL in the NSAID and opioid groups, respectively.

Opioids are generally used for postoperative pain management due to their strong analgesic effect. Thus far, most intravenous PCA regimens used in South Korea have comprised either opioids alone or with adjuvant analgesics [3,20,21]. NSAIDs are frequently administered as bolus injections for acute pain management, but their effects are less robust compared to opioids [22]. However, our analysis revealed that postoperative pain scores were similar between the opioid and NSAID groups, except for pain scores at 24 h postoperatively; the mean NRS scores were 3.8 and 4.0 in the NSAID and opioid groups, respectively. We measured pain scores using an 11-point NRS; this difference of 0.2 points seems to indicate a clinical similarity. These results suggest that NSAID PCA can provide adequate analgesia after laparoscopic nephrectomy.

Notably, the length of hospital stay was shorter in the NSAID PCA group than in the opioid PCA group. This finding is consistent with the results in a retrospective review of living kidney donors who underwent open nephrectomy, which showed that patients treated with NSAID-based analgesia could drink more fluids on the first postoperative day; moreover, they could more rapidly resume a regular diet and discontinue pain medications, resulting in a shorter mean hospital stay compared to patients treated with opioid-based analgesia [23].

This study has a few limitations. First, it did not assess several variables, such as postoperative nausea and vomiting, or the amount of analgesics consumed postoperatively, including those for PCA. Those variables could offer a clearer interpretation of our results. Second, a significant proportion of data was lost during the propensity score matching step. A further prospective randomized clinical trial is needed to better assess the applicability of NSAID PCA in clinical settings. Third, this study compared renal functions and postoperative outcomes in patients who had been treated with intravenous PCA. However, other PCA regimen types (e.g., epidural analgesia or other regional blocks) may also be opioid alternatives. Further studies are needed to compare the postoperative renal function and postoperative outcomes of those other analgesic strategies in patients undergoing laparoscopic nephrectomy.

## 5. Conclusions

In conclusion, this study suggests that there was no association between the use of NSAID PCA and postoperative renal dysfunction, and the length of hospital stay was shorter in the NSAID PCA group than in the opioid PCA group after laparoscopic nephrectomy.

## Figures and Tables

**Table 1:** Patient demographic characteristics and preoperative renal function test.

	NSAID PCA (*n* = 247)	Opioid PCA (*n* = 247)	*p*-Value
Age (Year)	53.6 ± 13.1	53.9 ± 12.5	0.762
Sex: Male	165 (66.8%)	168 (68%)	0.773
Height (cm)	165.8 ± 9.7	165.4 ± 8.8	0.584
Weight (kg)	69.2 ± 13.6	68.7 ± 12.2	0.652
BUN/Creatinine Ratio	18.1 ± 5.8	17.2 ± 5.0	0.116
Creatinine (mg/dL)	0.83 ± 0.2	0.84 ± 0.2	0.744
GFR (ml/min/1.73 m^2^)	94.8 ± 13.8	94.6 ± 14.9	0.828
Anemia	56 (22.7%)	50 (20.2%)	0.511
Smoking	126 (51%)	112 (45.3%)	0.207
Hypertension	81 (32.8%)	93 (37.7%)	0.258
Diabetes	18 (7.3%)	14 (5.7%)	0.465
CKD	0 (0%)	2 (0.8%)	0.156
CAD	6 (2.4%)	3 (1.2%)	0.313
Preoperative NSAIDs Use	14 (5.7%)	13 (5.3%)	0.843
Preoperative Opioids Use	12 (4.9%)	11 (4.5%)	0.831

NSAID, non-steroidal anti-inflammatory drug; PCA, patient-controlled analgesia; BUN, blood urea nitrogen; GFR, glomerular filtration rate; CKD, chronic kidney disease; CAD, coronary artery disease. Data are presented as mean ± SD or number (%).

**Table 2:** Anesthetic and surgical characteristics.

	NSAID PCA (*n* = 247)	Opioid PCA (*n* = 247)	*p*-Value
Robotic/Laparoscopic	227/20 (91.9%/8.1%)	219/28 (88.7%/11.3%)	0.244
Radical/Partial	45/202 (18.2%/81.8%)	51/196 (20.6%/79.4%)	0.495
Diagnosis			0.083
RCC	235 (95.1%)	222 (89.9%)	
Neoplasms Other Than RCC	10 (4.1%)	20 (8.1%)	
Other Disorders	2 (0.8%)	5 (2%)	
Primary Tumor Stage (pT)			0.245
1a	167 (67.6%)	159 (64.4%)	
1b	37 (15%)	35 (14.2%)	
2a	4 (1.6%)	9 (3.6%)	
2b	6 (2.4%)	1 (0.4%)	
2c	1 (0.4%)	1 (0.4%)	
3a	22 (8.9%)	24 (9.7%)	
4	0 (0%)	1 (0.4%)	
Resection Margin			0.021*
<0.1 cm	21 (8.5%)	8 (3.2%)	
0.1 to 0.5 cm	164 (66.4%)	161 (65.2%)	
≥0.5 cm	62 (25.1%)	78 (31.6%)	
Main Anesthetic Agent: Desflurane/Sevoflurane/Propofol	242/5/0 (98%/2%/0%)	238/8/1 (96.4%/3.2%/0.4%)	0.422
PCA Medication	Ketorolac (100%)	Fentanyl/Oxycodone (81%/19%)	
Anesthesia Time (min)	180.8 ± 180.8	184.6 ± 184.6	0.383
Crystalloid (mL)	1921.4 ± 663.7	1971.3 ± 695.3	0.414
Colloid (mL)	(*n* = 14) 721.5 ± 457.7	(*n* = 37) 562.6 ± 301.6	0.154
Transfusion (mL)	(*n* = 3) 956.7 ± 498	(*n* = 4) 552.5 ± 545	0.361
Urine (mL)	411.0 ± 301	444.1 ± 334	0.249
EBL (mL)	159.3 ± 285.2	172.5 ± 225.1	0.650
Intraop Hypotension (MBP < 55mmHg)	54 (21.9%)	62 (25.1%)	0.396

NSAID, non-steroidal anti-inflammatory drug; PCA, patient-controlled analgesia; RCC, renal cell carcinoma. Neoplasms other than RCC: angiomyolipoma, oncocytoma, mixed epithelial tumor, mesenchymal tumor and complicated renal cyst. Other disorders: ureteropelvic junctional obstruction, gangrenous pyelonephritis, and painful atrophic kidney. EBL, estimated blood loss; Intraop, intraoperative; MBP, mean blood pressure. Data are presented as mean ± SD or number (%). * *p*-value < 0.05.

**Table 3:** Postoperative renal function and postoperative outcomes.

	NSAID PCA (*n* = 247)	Opioid PCA (*n* = 247)	*p*-Value
GFR Percentage (%)			
2 Weeks	90.5 ± 16.0	89.5 ± 15.4	0.5
6 Months	90.9 ± 16.6	90.8 ± 14.9	0.946
1 Year	92.0 ± 15.0	90.2 ± 15.4	0.206
BUN/Creatinine Ratio			
2 Weeks	16.9 ± 5.5	17.2 ± 6.0	0.539
6 Months	17.4 ± 5.2	17.1 ± 5.2	0.53
1 Year	17.8 ± 5.3	17.4 ± 4.8	0.347
Creatinine Percentage (%)			
2 Weeks	129.5 ± 2.5	113.7 ± 0.2	0.337
6 Months	118 ± 0.8	113.7 ± 0.2	0.424
1 Year	110.3 ± 0.2	113.7 ± 0.2	0.093
Clavien–Dindo Complication			0.885
1	154 (62.3%)	150 (60.7%)	
2	1 (5.7%)	13 (5.3%)	
3A	2 (0.8%)	1 (0.4%)	
AKI			0.425
Stage 1	22 (8.9%)	16 (6.5%)	
Stage 2	0 (0%)	3 (1.2%)	
Stage 3	2 (0.8%)	0 (0%)	
Total	24 (9.7%)	19 (7.7%)	
Pain score (NRS)			
30 min	6.0 ± 1.6	5.8 ± 2.0	0.268
6 h	4.3 ± 0.8	4.3 ± 1.0	0.514
24 h	3.8 ± 0.8	4.0 ± 0.9	0.010 *
48 h	3.6 ± 0.7	3.7 ± 0.8	0.246
7 Days	2.2 ± 0.8	2.1 ± 0.9	0.458
Numbers of PCA-Use Days	3.2 ± 1.0	3.9 ± 1.2	0.000 *
Length of Hospital Stay (Days)	7.5 ± 0.8	8.5 ± 0.9	0.000 *

NSAID, non-steroidal anti-inflammatory drug; PCA, patient-controlled analgesia; GFR, glomerular filtration rate; GFR percentage means postoperative GFR divided by preoperative GFR; BUN, blood urea nitrogen; AKI, acute kidney injury; NRS, numerical rating scale. Data are presented as mean ± SD or number (%). * *p*-value < 0.05.
